# *Bacopa monnieri* extract increases rat coronary flow and protects against myocardial ischemia/reperfusion injury

**DOI:** 10.1186/s12906-017-1637-z

**Published:** 2017-02-20

**Authors:** Sirintorn Srimachai, Sylvie Devaux, Celine Demougeot, Sarawut Kumphune, Nina D. Ullrich, Ernst Niggli, Kornkanok Ingkaninan, Natakorn Kamkaew, C. Norman Scholfield, Sompol Tapechum, Krongkarn Chootip

**Affiliations:** 1 Department of Physiology, Faculty of Medicine, Siriraj Hospital, Mahidol University, Bangkok, 10700 Thailand; 2 Department of Physiology, Faculty of Medical Science, Naresuan University, Phitsanulok, 65000 Thailand; 3 PEPITE EA4267, University Bourgogne Franche-Comté, Besançon, F-25000 France; 4 Department of Medical Technology, Faculty of Allied Health Sciences, Naresuan University, Phitsanulok, 65000 Thailand; 5 Biomedical Research Unit in Cardiovascular Sciences, Faculty of Allied Health Sciences, Naresuan University, Phitsanulok, 65000 Thailand; 6 Department of Physiology, University of Bern, Bühlplatz 5, Bern, CH-3012 Switzerland; 7 Department of Physiology and Pathophysiology, Heidelberg University, Heidelberg, 69120 Germany; 8 Department of Pharmaceutical Chemistry and Pharmacognosy, Faculty of Pharmaceutical Sciences, Naresuan University, Phitsanulok, 65000 Thailand; 9 School of Medical Sciences, University of Phayao, Phayao, 56000 Thailand

**Keywords:** *Bacopa monnieri*, Brahmi, Heart, Coronary blood flow, Ischemia/reperfusion, Cardiac function, Myocardial infarction

## Abstract

**Background:**

This study explored *Bacopa monnieri*, a medicinal Ayurvedic herb, as a cardioprotectant against ischemia/reperfusion injury using cardiac function and coronary flow as end-points.

**Methods:**

In normal isolated rat hearts, coronary flow, left ventricular developed pressure, heart rate, and functional recovery were measured using the Langendorff preparation. Hearts were perfused with either (i) Krebs-Henseleit (normal) solution, (control), or with 30, 100 μg/ml *B. monnieri* ethanolic extract (30 min), or (ii) with normal solution or extract for 10 min preceding no-perfusion ischemia (30 min) followed by reperfusion (30 min) with normal solution. Infarct volumes were measured by triphenyltetrazolium staining. L-type Ca^2+^-currents (I_Ca, L_) were measured by whole-cell patching in HL-1 cells, a mouse atrial cardiomyocyte cell line. Cytotoxicity of *B. monnieri* was assessed in rat isolated ventricular myocytes by trypan blue exclusion.

**Results:**

In normally perfused hearts, *B. monnieri* increased coronary flow by 63 ± 13% (30 μg/ml) and 216 ± 21% (100 μg/ml), compared to control (5 ± 3%) (*n* = 8–10, *p* < 0.001). *B. monnieri* treatment preceding ischemia/reperfusion improved left ventricular developed pressure by 84 ± 10% (30 μg/ml), 82 ± 10% (100 μg/ml) and 52 ± 6% (control) compared to pre- ischemia/reperfusion. Similarly, functional recovery showed a sustained increase. Moreover, *B. monnieri* (100 μg/ml) reduced the percentage of infarct size from 51 ± 2% (control) to 25 ± 2% (*n* = 6-8, *p* < 0.0001). *B. monnieri* (100 μg/ml) reduced I_Ca, L_ by 63 ± 4% in HL-1 cells. Ventricular myocyte survival decreased at higher concentrations (50–1000 μg/ml) *B. monnieri*.

**Conclusions:**

*B. monnieri* improves myocardial function following ischemia/reperfusion injury through recovery of coronary blood flow, contractile force and decrease in infarct size. Thus this may lead to a novel cardioprotectant strategy.

**Electronic supplementary material:**

The online version of this article (doi:10.1186/s12906-017-1637-z) contains supplementary material, which is available to authorized users.

## Background

Disease of the myocardial vasculature including the coronary artery is a major cause of disability and death in spite of widespread application of highly efficacious cardiovascular drugs. Myocardial infarction (MI) most commonly arises from an unstable atheroma forming fragments and clots that occlude downstream vessels culminating in regional myocardial ischemia [1]. The resultant energy failure has numerous cytopathological consequences, including changes of pH and Na^+^ levels, subsequently leading to reversal of Na^+^/Ca^2+^ exchange, which in turn results in cytosolic Ca^2+^ loading and activation of proteolytic enzymes mediating necrosis [2]. Mitochondrial Ca^2+^ homeostasis also fails, leading to disruption of the electron transport chain, cytochrome C release and initiation of apoptosis. However, these changes are slow during sustained occlusion [2] but most damage begins on reperfusion. Glucose is shunted into glycolysis producing lactic acid and mitochondrial beta-fatty acid oxidation [2]. Early reperfusion is accompanied by excessive superoxide production [3] and consequently leads to other reactive species which increase cell damage and to further cytosolic Ca^2+^ loading [4]. Fragments of damaged cells then activate resident immune cells and immune receptors on endothelial cells, which in turn secrete inflammatory cytokines and adhesion proteins, respectively. Endothelial cells generate further superoxide via adherent xanthine oxidase, cytokine activation of nicotinamide adenine dinucleotide phosphate (NADPH) oxidase, and oxidised nitric oxide (NO) synthase [5]. Endothelial cells not only lose their vasorelaxant influence over vascular smooth muscle, but they release vasoconstrictors, especially endothelin-1 [6], which further exacerbates the conditions causing MI. Endothelial injury manifests as cell blebbing, swelling and plugging culminates in “no-reflow” [7]. Thus we have a potent cytotoxic combination of Ca^2+^, superoxide, inflammation and vasoconstriction, all of which conspire over the ensuing minutes-hours in myocardial cell death.

Targeting these pathologies individually produces improved recoveries in experimental animals provided the protectant is present before ischemia begins, but they have failed in translation. Thus at present, the standard treatments focus on atherosclerosis by using thrombolytics, anticoagulants or angioplasty. Clinical applications targeting mitochondria have met with some limited positive outcomes [2, 8]. Nevertheless, it is clear that for any protectant to be useful, it would have to be administered prophylactically to high risk patients, act against the major reperfusion pathologies, be cheap and have low toxicity and ideally, help to reduce the initial risk of MI. It is unlikely that any classical single substance or drug could perform in this manner, but several multifunctional herbals have the potential to fulfill such a role.

They include, a medicinal Ayurvedic herb, which is thought to improve cardiovascular function. Thus, oral *B. monnieri* given to rats for three weeks reduced biochemical and histopathological perturbations caused by ischemia/reperfusion (I/R), while increasing myocardial antioxidant enzymes and reducing myocardial apoptotic signaling proteins [9]. Over-dosing rats with isoproterenol reduced endogenous antioxidant enzymes and increased lipid peroxidation, but less so in those animals which had been chronically treated with *B. monnieri* [10]*.* Necrosis and markers of myocardial injury were also reduced.

There is also some evidence that *B. monnieri* improves systemic vascular function. It relaxes a wide range of arteries [11], partly through endothelial NO release and in vascular smooth muscle by inhibiting Ca^2+^ influx and Ca^2+^ release from the sarcoplasmic reticulum [11]. Nevertheless, chronic oral administration of *B. monnieri* to rats for 12 weeks increased cerebral blood flow without changing blood pressure [12]. This is particularly important because *B. monnieri* has been traditionally used to enhance memory and several clinical trials suggest that it does have nootropic actions [13, 14]. Furthermore, it is neuroprotective [15, 16] and the mechanism of action is mainly related to its antioxidant and anti-inflammatory properties [16–18]. Recently, we have shown that *B. monnieri* had no signs of toxicity in either acute or chronic oral toxicity tests of both male and female rats, indicating that it is relatively safe [19].

Clearly, several lines of evidence point to *B. monnieri* having potentially favorable cardiovascular actions arising from the hemodynamic changes and protective effects in vulnerable tissues as well as reducing some of the pathological changes contributing to I/R injury. Nevertheless, it is unclear whether these effects of *B. monnieri* translate directly to improving the function of the myocardium or whether they could ameliorate the damage following cessation and restoration of blood flow, i.e., I/R injury. To answer this, we aimed to show that an extract of *B. monnieri* could improve myocardial perfusion in normally beating isolated rat hearts and to show that it produces an independent acute recovery in cardiac function after I/R while at the same time not creating cellular toxicity in isolated ventricular myocytes.

## Methods

### Preparation of *B. monnieri* extract

The aerial part of *B. monnieri* was collected from Phetchaburi province, Thailand, and identified by Associate Professor Wongsatit Chuakul, Faculty of Pharmacy, Mahidol University, Thailand. The voucher specimen (Phrompittayarat 001) was kept at the Pharmaceutical Botany Mahidol Herbarium, Mahidol University, Thailand. *B. monnieri* was extracted using 95% ethanol and its total saponin content, 4.19% (w/w) comprising bacoside A_3_ (0.74%), bacopaside I (0.93%), bacopaside II (0.98%) bacopaside X (0.53%), and bacopasaponin C (1.00%), was determined by high pressure liquid chromatography as previously reported [20, 21]. The extract was dried and stored at 4 °C until use. Its yield was 10% (w/w of the dried plant). The *B. monnieri* extract was dissolved in Krebs-Henseleit buffer (KHB) (mM): NaCl, 118; NaHCO_3_, 25; KH_2_PO_4_, 1.2; KCl, 4.5; CaCl_2_, 1.36; MgSO_4_, 1.2 and glucose 11 adjusted to pH 7.4. It was stirred for 30 min and sonicated in an ultrasonic tank at 37 °C for another 30 min. Prior to use, the KHB and the solutions of *B. monnieri* extract were filtered through a 0.8 μm Millipore filter to remove insoluble particles.

### Animals

Adult male Wistar rats (200–250 g, 7–8 weeks old) were obtained from the National Laboratory Animal Centre, Mahidol University, Salaya, Nakhorn Pathom, Thailand or Charles River, L’Arbresle, France. All experimental protocols were approved by the Animal Ethics Committee (No. NU-AE530613 and NU-AE590203), Naresuan University, Phitsanulok, Thailand and by the local committee for ethics in animal experimentation (No. 2012-012-CD, date 05/09/2012), Faculty of Medicine and Pharmacy, Université de Franche Comte, Besançon, France, and complied with the US National Institutes of Health (NIH publication No. 85–2, revised 1996). Rats were kept in polysulfone shoe box cage (3–4 rats/cage) bedded with autoclaved corn cob, in temperature-controlled rooms (22 ± 2 °C), at 50-60% humidity, with a reversed 12:12 h diurnal cycle and standard diet and RO water provided *ad libitum*. Animals were randomly allocated for ex vivo treatment. The sample size was calculated using R program (α = 5%, mean difference = 15, SD = 10, power = 80%).

### Preparation of Langendorff heart perfusion and protocols

#### Effect of *B. monnieri* on normal and I/R hearts

Rats were anesthetized with sodium pentobarbital (50 mg/kg, intraperitoneally) followed by heparin (250 UI/kg, intravenously). The level of anesthesia was assessed by toe pinch and corneal reflex before the surgery. The hearts were then rapidly removed and immersed in ice-cold KHB to stop contractions, and the myocardium was perfused retrogradely with KHB using the Langendorff method at a constant perfusion pressure of 80 cm of water (7.8 kPa). The perfusate was bubbled with 95% oxygen and 5% carbon dioxide and maintained at 37 °C. To measure left ventricular end diastolic pressure (LVEDP), a water-filled latex balloon (4x8 mm, EMKA Technologies, Paris, France) was inserted into the left ventricle and connected to a pressure transducer (682002, Becton Dickinson, Sandy, Utah, USA) and a Gould TA240 Physiograph (Gould Instrument Systems, Courtaboeuf, France). The pressure within the balloon was adjusted to 2–8 mmHg, i.e., equivalent to the normal LVEDP [22].

The intraventricular pressure and heart rate (HR) were continuously recorded. The left ventricular developed pressure (LVDP) calculated as left ventricular systolic pressure minus LVEDP, was used as an indicator of left ventricular systolic function. Total coronary flow was measured by collecting aliquots of coronary venous effluent. All hemodynamic parameters were measured at 2 min intervals. The hearts were stabilized for 10–20 min and then the following two experimental protocols (Table 1) were carried out (54 rats*, n* = 8-10/group).

**Table 1:** Experimental design of *B. monnieri* effect on normal and I/R hearts

Group	Treatment & Protocol
Normal hearts	1) KHB (control, *n* = 9)
	2) 30 μg/ml *B. monnieri* extract (*n* = 10)
	3) 100 μg/ml *B. monnieri* extract (*n* = 8)
	Protocol: Isolated hearts were perfused with KHB alone or KHB containing *B. monnieri* extract for 30 min (Fig. 1).
I/R hearts	1) KHB (control, *n* = 9)
	2) 30 μg/ml *B. monnieri* extract (*n* = 9)
	3) 100 μg/ml *B. monnieri* extract (*n* = 9)
	Protocol: Isolated hearts were perfused with KHB alone or KHB containing *B. monnieri* extract for 10 min and then perfusion stopped for 30 min to emulate global myocardial ischemia, after which 30 min perfusion was resumed with normal oxygenated KHB (Fig. 2).

The concentrations of *B. monnieri* extract were based on earlier in vitro studies where the IC_50_ was around 100 μg/ml for inducing vasorelaxation in small resistance arteries [11].

#### Effect of *B. monnieri* on myocardial infarct size

Rat hearts were isolated and perfused using the Langendorff method as described above. The hearts were pre-perfused with KHB (control) or KHB supplemented with 100 μg/ml *B. monnieri* for 30 min prior to 30 min global ischemia followed by 2 h reperfusion, sufficient to allow histological changes to develop [23] (14 rats, *n* = 8 for control, *n* = 6 for treatment). After 2 h of reperfusion, hearts were perfused for 5 min with 10 ml of 1% (w/v) triphenyltetrazolium chloride (TTC) (Sigma, St. Louis, MO, USA), then removed and placed in 1% TTC solution at 37 °C for 10 min [23]. The hearts were incubated in 2.5% glutaraldehyde for 1 min, and set in 5% agarose before sectioning in 750 μm thick slices. All slices were incubated in 10% (v/v) formaldehyde overnight at room temperature before re-hydration overnight with phosphate buffer saline (PBS) at 4 °C. The heart sections were scanned and planimetry was carried out and surface area of the whole, and TTC-negative, myocardium was transformed to volume [23]. The TTC-negative infarction volume was expressed as a percentage of heart volume. All analyses of infarct size were done by an investigator, who was blinded with regard to the group assignments.

### Electrophysiological measurements of the L-type Ca^2+^ current

Effect of *B. monnieri* (100 μg/ml) on L-type Ca^2+^ current (I_Ca, L_) was studied in HL-1 cells (kind gift from Prof. Dr. William C. Claycomb, Louisiana State University Medical Center, LA, USA). This cell line, derived from an atrial tumor lineage of the AT-1 mouse, spontaneously contracts and retains the phenotypic characteristics of cardiomyocytes [24], including the expression of L-type Ca^2+^ channels. Inhibition of I_Ca, L_ by nifedipine was previously demonstrated in HL-1 cells as shown in Additional file 1: Figure S1. The whole-cell patch clamp technique was used for recording of I_Ca, L_. The experimental protocol was adopted from a previous study [25]. The composition of the bath solution (i.e., normal Tyrode’s solution) was (mM): NaCl, 140; KCl, 5.4; MgCl_2_, 1.1; CaCl_2_, 2; HEPES, 5; glucose, 10 and adjusted pH to 7.4, osmolality, 310 mOsm. Patch pipettes were fabricated from capillary glass tubing (Borosilicate-standard wall with filament, 1.50 mm OD). Internal solution was composed of (mM): NaCl, 8; MgCl_2_, 5.9; HEPES, 20; Cs aspartate, 120; TEA-Cl, 20; K_2_-ATP, 5 (pH 7.2, osmolality, 290 mOsm). The pipette resistance was 3–5 MΩ in the bath solution. Cells were locally perfused with Tyrode’s solution plus CsCl_2_ (5 mM) to block K^+^ channels. I_Ca, L_ was recorded using following protocols [25]: holding membrane potential was kept at −80 mV. A pre-step to −40 mV for 1500 ms inactivated Na^+^ and T-type Ca^2+^ channels, then a test-step to +10 mV for 400 ms was applied to measure L-type Ca^2+^ currents. Control I_Ca, L_ was recorded 3 times to obtain consistency of the current of the cell chosen. Then, each cell was locally perfused with 100 μg/ml *B. monnieri* extract dissolved in Tyrode’s solution containing CsCl_2_ for 1 min. Subsequently, currents were recorded in the presence of the extract. Current signals were recorded with an Axopatch 200B patch-clamp amplifier (Axon Instruments, Foster City, CA) connected to a 50/60 Hz Noise Eliminator (Hum Bug, Quest Scientific, Canada). Data were collected using a custom-written program (Myoclamp V2.4.1 software) for Windows. Currents were analyzed by using IGOR Pro6 software (Wave-Metrics). The average effect of the extract on I_Ca, L_ was expressed as percentage change of control I_Ca, L_ from the same cell.

### Cytotoxicity of *B. monnieri*

#### Isolation of rat ventricular myocytes and culture

Ventricular myocytes were isolated from rat hearts using collagenase as previously described [23, 26]. Hearts were excised and initially perfused in the Langendorff apparatus for 5 min with Tyrode’s solution containing (mM): NaCl, 130; KCl, 4.5; MgCl_2_ 1.4; NaH_2_PO_4_, 0.4; CaCl_2_, 0.75; [N-(2-hydroxyethyl) piperazine N′-(2-ethanesulfonic acid)] HEPES 4.2, taurine 20, creatine 10, glucose 10, pH 7.3 and gassed with 100% O_2_ at 37 °C and then perfused with Ca^2+^ free Tyrode’s solution containing 100 μM EGTA for another 4 min. This was followed by Tyrode’s solution containing 100 μM CaCl_2_ and 1 mg/ml Worthington type II collagenase for 8 min. The ventricles were then cut into approximately 1 mm^3^ pieces and incubated in 10 ml of this collagenase solution for 7 min with regular triturating using a Pasteur pipette until cells were released. Isolated myocytes were separated from undigested ventricular tissue by filtering through cell strainer. Isolated myocytes were allowed to settle into a loose pellet and the supernatant was removed and replaced with Tyrode’s solution containing 1% fetal bovine serum albumin and 500 μM CaCl_2_. In the next step, the supernatant was removed and replaced with Tyrode’s solution containing 1 mM CaCl_2_. The cell pellet was washed at room temperature with M199 culture medium containing 100 IU/ml penicillin/streptomycin. The myocytes were resuspended in modified M199 (containing 2 mM creatine, 2 mM carnitine, and 5 mM taurine).

### Determination of cell viability

Cell viability was assessed by trypan blue exclusion assay [26]. After cell plating, the isolated cardiac myocytes were replenished with modified M199 culture medium (control) or medium containing *B. monnieri* extract (10–1000 μg/ml) for 1 h. Then the supernatants were removed, the cells were resuspended in 50 μl 0.4% trypan blue solution and incubated for 5 min. After that, supernatants were removed and replaced with PBS solution. One hundred cells in each well (*n* = 3) were randomly counted under a light microscope. The cell viability was calculated as number of unstained cells divided by total number of cells and expressed as a percentage.

### Drugs and solution

All chemicals were purchased from Sigma (St. Louis, MO, USA or Saint Quentin Fallavier, France). M199 medium and fetal bovine serum were from Gibco BRL, Life Technologies, Inc. (New York, USA)*.* Type II collagenase was obtained from Worthington (Lakewood, NJ, USA).

### Statistical and data analysis

All data are presented as mean ± standard error of mean (SEM) of *n* animals or cells. Comparisons were assessed using analysis of variance (two-way ANOVA) followed by Tukey’s test or Student *t*-test as appropriate. Statistical significance was considered at *p* values <0.05. To compare the effects of *B. monnieri* with controls, all hemodynamic parameters were normalized to the data value obtained after the 10 min stabilization period, and were expressed as %coronary flow, %LVDP, %HR, and %functional recovery, a global index of cardiac contractile function, calculated as [(HR TRx × LVDP TRx × 100) / HR TP10 × LVDP TP10] where TRx represents the time of reperfusion at x min and TP10 relates to the value at 10 min of the perfusion during stabilization period.

## Results

### *B. monnieri* increased coronary flow in normal hearts

**Fig. 1:**
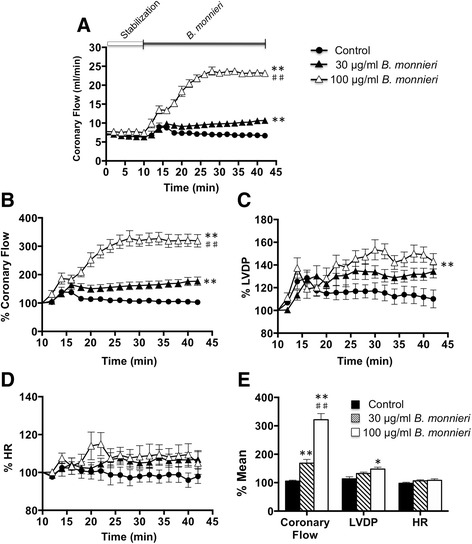
*B. monnieri* improved hemodynamics in isolated rat hearts. **a** coronary flow (ml/min) using normal perfusate (control) and perfusate containing 30 or 100 μg/ml *B. monnieri* extract for 30 min, **b** coronary flow, %, **c** LVDP, %, **d** HR, %: **b**-**d** expressed as percentage of mean data from the same heart compared to the 10 min stabilization using normal perfusate and **e** mean expressed as the average of each parameter after application of normal perfusate or perfusate containing *B. monnieri* extract for 15 min onward. Data are expressed as mean ± SEM (*n* = 8-10 hearts) and *p < 0.05, **p < 0.001 for 30, 100 μg/ml *B. monnieri* compared to control, ^##^ *p* < 0.001 for 30 μg/ml compared to 100 μg/ml *B. monnieri*

In normally perfused isolated rat hearts, during stabilization, all hemodynamic parameters including coronary flow (Fig. 1a), LVDP and HR remained constant and similar in all treatment groups. Application of 30 or 100 μg/ml *B. monnieri* for 30 min produced a concentration-dependent increase in coronary flow (Fig. 1b) and LVDP (Fig. 1c). The coronary flow was 1.6-fold increased in the presence of *B. monnieri* at 30 μg/ml (mean = 168 ± 13%, *n* = 10), and 3.1-fold at 100 μg/ml (mean = 321 ± 21%, *n* = 8) compared to control (mean = 105 ± 3%, *n* = 9) (Fig. 1e). There were also small increases in LVDP (Fig. 1c, e), but no change in HR (Fig. 1d, e) in response to both concentrations of *B. monnieri*.

### *B. monnieri* improved recovery after I/R

**Fig. 2:**
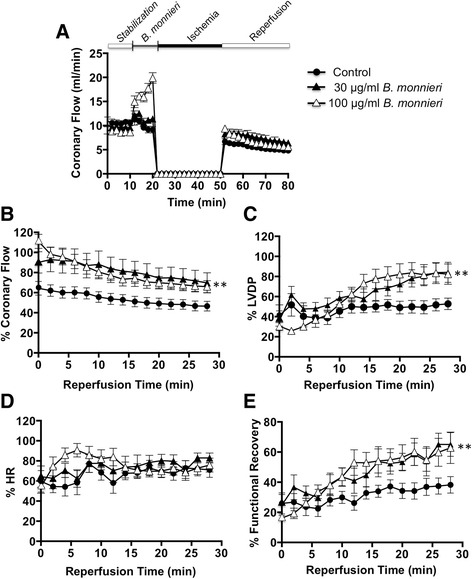
Pretreatment with *B. monnieri* and recovery after myocardial ischemia in isolated rat hearts. **a** coronary flow (ml/min) using normal perfusate (control) or perfusate containing 30 or 100 μg/ml *B. monnieri* (10 min), followed by cessation of perfusion (30 min, ischemia) and then restoration of normal perfusate (30 min, reperfusion). **b**-**e** truncated time courses showing the post-ischemia recovery period where the values are expressed as percentage of the mean data from the same heart compared to the 10 min stabilization using normal perfusate for **b** coronary flow, %, **c** LVDP, %, **d** HR, % and **e** functional recovery, %, a global index of cardiac contractile function, calculated as [(HR TRx × LVDP TRx × 100) / HR TP10 × LVDP TP10] where TRx represents the time of reperfusion at x min and TP10 relates to the value at 10 min of the perfusion during stabilization period. Data are expressed as mean ± SEM (*n* = 9 hearts) and **p < 0.001 for 30 or 100 μg/ml *B. monnieri* compared to control

*B. monnieri* extract was added to the perfusate for 10 min whereupon coronary flow increased as above. The perfusion was then stopped for 30 min to emulate global ischemia after which perfusion was restored for a further 30 min (Fig. 2a). In the control group (*n* = 9), all the hemodynamic parameters were substantially reduced compared to before ischemia (Fig. 2b-e). However, pretreatment with 30 or 100 μg/ml *B. monnieri* improved cardiac function in I/R heart. Coronary flow was immediately restored to 90 ± 10% (*n* = 9), and 111 ± 7% (*n* = 9) with pre-exposure to 30 and 100 μg/ml *B. monnieri*, respectively (Fig. 2b). Although coronary flow declined, this was always higher in the *B. monnieri* pre-treated groups at every time point compared to those without *B. monnieri* (*p* < 0.001). LVDP as a measure of myocardial contractile force, gradually increased in *B. monnieri*-treated groups and maintained after reperfusion for 20 min onwards (52 ± 6%, 84 ± 10%, 82 ± 10%, for control, 30 μg/ml and 100 μg/ml *B. monnieri*, respectively, *p* < 0.001 for *B. monnieri* compared to control) (Fig. 2c). Likewise, functional recovery showed a sustained increase (Fig. 2e). HR was depressed on initial reperfusion, but there was no clear change during the recovery period (Fig. 2d).

### Pretreatment of *B. monnieri* reduced myocardial infarction

**Fig. 3:**
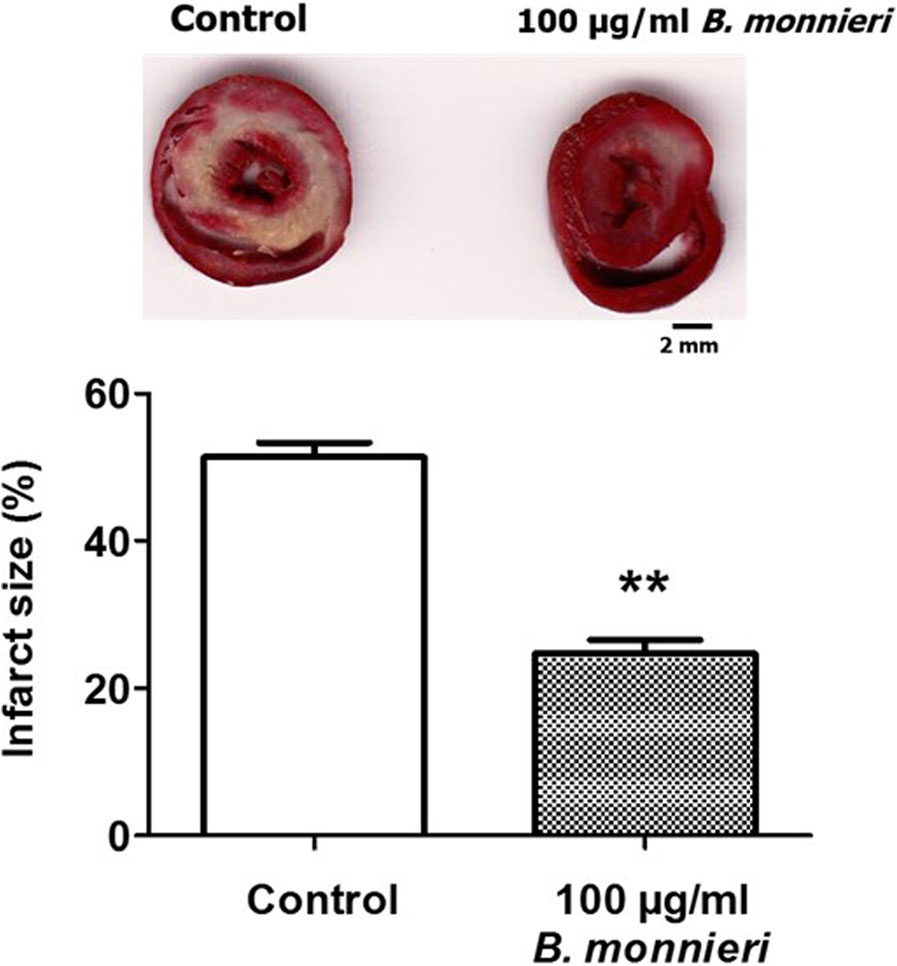
The sensitivity to myocardial infarction in rat hearts subjected to global ischemia/reperfusion, in the presence or absence of 100 μg/ml *B. monnieri*. The data shown is the normalised infarction volume of isolated rat hearts in the presence of 100 μg/ml *B. monnieri* extract for 30 min before the onset of 30-min global ischemia followed by 2 h of reperfusion. The bar graphs represent means ± SEMs (*n* = 8 for control and *n* = 6 for *B. monnieri* extract, **p < 0.0001 *vs* control)

An ex vivo global ischemia for 30 min followed by 2 h reperfusion resulted in the myocardial infarction up to 51 ± 2% of ventricular volume (*n* = 8) (Fig. 3, control). Pretreatment with 100 μg/ml *B. monnieri* substantially reduced infarct size to 25 ± 2%, *n* = 6 (Fig. 3, *p* < 0.0001 *vs* control).

### *B. monnieri* reduced I_Ca, L_ in HL-1 cardiac cells

Ca^2+^ is an important mediator of ischemic cell damage and in cardiac muscle, an important Ca^2+^ influx pathway is through L-type Ca^2+^ channels and blockade of these channels can offer some protection [27, 28].

**Fig. 4:**
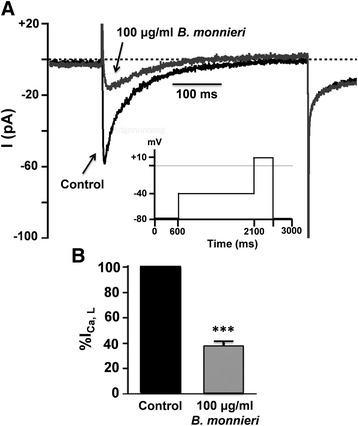
For whole cell currents in voltage clamped HL-1 cardiac cells, K^+^-currents were blocked by intracellular and extracellular Cs^2+^, and Na^+^- and T-currents inactivated by a -40 mV holding potential, then I_Ca, L_ activated by stepping to +10 mV (voltage profile: see inset). **a** Typical trace showing inhibition of I_Ca, L_ by 100 μg/ml *B. monnieri* in the same cell. **b** Inhibition by 100 μg/ml *B. monnier* of I_Ca, L_ expressed as percentage of mean compared to average of control I_Ca, L_ from the same cell. Data are means ± SEM (*n* = 4 cells from 3 cultures) and *** *p* < 0.001 comparing *B. monnieri vs* control, paired *t*-test

Whole-cell voltage clamping of HL-1 cardiac cells showed transient inward currents of −71.4 ± 19.6 pA previously identified as I_Ca, L_ [25] (‘control’ in Fig. 4a). The extract decreased the peak current amplitude of I_Ca, L_ to about one third i.e., to 37.1 ± 4.1% compared to control (*p* < 0.001, *n* = 4, Fig. 4b).

### Cytotoxicity of *B. monnieri* extract

**Fig. 5:**
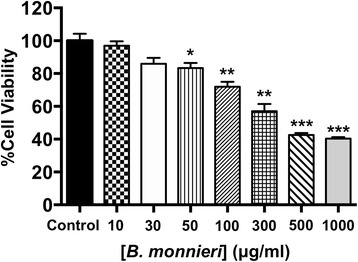
Toxicity testing of *B. monnieri* extract (10–1000 μg/ml) for 1 h on rat freshly isolated ventricular myocyte viability quantified by the trypan blue exclusion assay. Data are expressed as mean ± SEM (*n* = 3) and *p < 0.05, **p < 0.01 ***p < 0.001 for *B. monnieri* compared to control

Direct effect of *B. monnieri* on cell viability of freshly isolated rat ventricular myocytes was determined using the trypan blue exclusion assay. After 1 h incubation of ventricular myocytes with various concentrations of *B. monnieri* extract (10–1000 μg/ml), cell survival was decreased in those cells exposed to concentrations of 50 μg/ml and above (Fig. 5). High concentrations of the extract (≥500 μg/ml) produced more than 50% cell death (Fig. 5).

## Discussion

The key findings of the present study were that *B. monnieri* extract induced a concentration-dependent improvement in coronary flow, promoted cardiac function, and reduced infarct area resulting from ischemia and reperfusion in isolated perfused hearts. Previously, *B. monnieri* was shown to dilate various isolated rat arteries by (i) stimulating endothelial NO release, and (ii) acting directly on vascular smooth muscle cells by inhibiting extracellular Ca^2+^ influx and Ca^2+^ release from sarcoplasmic reticulum [11]. Also, *B. monnieri* inhibits Ca^2+^ influx in various tissues including several types of isolated rat arteries [11, 29], guinea pig ileum and trachea [29, 30] and rat trachea [31]. Similar mechanisms may well operate in the coronary vasculature. Likewise, chronic oral administration of *B. monnieri* increased cerebral blood flow in normal rats [12].

Coronary blood flow is normally mediated by demands of myocardial contraction [32]. However, in the presence of *B. monnieri* coronary flow was substantially greater than the increased LVDP, which suggests that the vasculature dilated independently of myocardial needs. Nevertheless, there was a slightly increased myocardial contractility with *B. monnieri*, which might be facilitated by the augmented blood flow as seen in the canine and rat hearts [33, 34]. Moreover, the positive ionotropic effect of *B. monnieri* suggests the extract had no adverse action on the intact myocardium.

However, there was a clear disconnect between blood flow and contractility during post-ischemic reperfusion: restoration of blood flow was immediate and slowly declined thereafter while LVDP developed over 15 min (Fig. 2). This implies that contractility was not solely a direct consequence of better blood flow. One explanation for this disconnect could involve Ca^2+^ influx, which we show was inhibited in isolated myocytes. On the one hand, inhibition of vascular smooth muscle L-type Ca^2+^ maximises the vasodilatation through the lingering action of *B. monnieri*, thus providing metabolic resources to re-establish the extra- and intracellular milieu. At the same time, this same action on cardiac myocytes inhibits their contraction until the accumulated Ca^2+^ is cleared after which they can re-establish sinus rhythm. This Ca^2+^ overloading of cardiac myocytes has been emphasised in many studies and various drugs targeting this overload including Ca^2+^-channel blockers improve post-IR recovery [27, 28].

Our present protocol was short-term but *B. monnieri* may well have more lasting beneficial effects in vivo [9, 10]. For example, in coronary I/R, there is inflammation beginning with endothelial cell blebbing and swelling followed by platelet and leucocyte adhesion leading to microvascular block and a “no flow” condition lasting many hours [7]. With *B. monnieri*, the initial flow was good but gradually decreased with continued reperfusion for all three treatment groups although the pretreated ones maintained superior flow rates (Fig. 2). The prolonged effects of inflammation do not apply to the present protocol, but our data suggest that the coronary vasculature enters this critical period with a distinct functional advantage. These anti-inflammatory effects of *B. monnieri* may be mediated through one of its constituents, betulinic acid [35, 36] by potently blocking the inflammatory transcription factor NF-kB or p38/ERK MAPK pathways [35] thereby preventing the augmented cytokine production [36].

Antioxidant actions are universally claimed to be an action of most herbals including *B. monnieri* [9, 17]. Yet, whatever of this antioxidant remained in the tissue on reperfusion without extract would have little real impact on the superoxide onslaught. Nevertheless, polyphenols and other plant biologicals stereospecifically cause Nrf-2 nuclear translocation thereby increasing expression of some anti-oxidant enzymes [37–40] and their activation can provide protection against I/R [41].

Our *B. monnieri* extract appeared to be cytotoxic at a threshold concentration of ~50 μg/ml but, nevertheless, it increased LVDP suggesting that toxicity was absent in the intact myocardium. The explanation for this discrepancy may be a combination of: (i) toxicity was studied in culture where myocytes are directly exposed to the extract, while in the perfused heart the concentration of the extract within the extracellular space is likely to be lower, (ii) the incubation time of cultured myocytes in the extract was longer (1 h) than the 10 min perfusion. Importantly, the intriguing notion is that if those components of *B. monnieri* having beneficial and having cytotoxic actions are different, the cardioprotective components could display a greater efficacy for post-ischemic recovery.

This study has revealed an improved coronary flow, increased myocardial function and contracted infarct area. The additional anti-inflammatory, antioxidant defenses and reduced Ca^2+^-overload demonstrated in other systems, suggest that *B. monnieri* might provide a multipronged defense against ischemic cell death. Nevertheless, the model used here, and most of the animal models for I/R poorly replicate the human condition where the underlying vascular pathology develops over a generation and is influenced by ‘conditioning’. Thus, major impacts in the treatment of I/R will only become translatable when accurate animal models for the human condition emerge.

## Conclusion

*B. monnieri* protected against I/R injury in rat heart as judged by its ability to recover coronary flow, contractile force and function, and reduced infarct volume. As such, it could form the basis of a promising cardioprotectant (and cerebroprotectant) for patients at risk from infarcts. In pursuing this, the protectant and cytotoxic components need to be identified and plant strains or extraction protocols showing more efficacious chemical profiles selected.

## Additional file

Additional file 1: Figure S1. Effect of L-Type Ca^2+^ Channel Blocker, Nifedipine, on I_Ca, L_ current. (DOCX 118 kb)

## Abbreviations

*B. monnieri*: *Bacopa monnieri*
HR: Heart rate
I/R: Ischemia/reperfusion
KHB: Krebs-Henseleit buffer
LVDP: Left ventricular developed pressure
LVEDP: Left ventricular end diastolic pressure
MI: Myocardial infarction
NADPH: Nicotinamide adenine dinucleotide phosphate
NO: Nitric oxide
PBS: Phosphate buffer saline
TP10: The value at 10 min of the perfusion during stabilization period
TRx: Time of reperfusion at x min
TTC: Triphenyltetrazolium chloride
