# Supplementary material for: Bacopa monnieri extract increases rat coronary flow and protects against myocardial ischemia/reperfusion injury
Source: BMC Complement Altern Med. 2017 Feb 20;17:117. doi: 10.1186/s12906-017-1637-z (PMC5319078; doi:10.1186/s12906-017-1637-z)
Supplement: Additional file 1: Figure S1. — Effect of L-Type Ca2+ Channel Blocker, Nifedipine, on ICa, L current. (DOCX 118 kb) [file 12906_2017_1637_MOESM1_ESM.docx]

**Supplementary Material**

**Effect of L-Type Ca^2+^ Channel Blocker, Nifedipine, on I_Ca, L_ current**

The HL-1 cell line used in the present study was derived from an atrial tumor lineage of the AT-1 mouse. It spontaneously contracts and retains the phenotypic characteristics of cardiomyocytes (see reference No. 24), including the expression of L-type Ca^2+^ channels. It is well documented that the patch clamp protocol used in this study is for determining the L-type Ca^2+^ current in HL-1 cells, since at the clamped pre-step potential to -40 mV, Na^+^ and T-type Ca^2+^ channels will be in their inactivated state. Figure below shows inhibition of the L-type Ca^2+^ current by nifedipine. The whole-cell patch clamp protocol used for recording of I_Ca, L_ was adopted from a previous study (see reference No. 25). Detail of protocol was explained in the methods section.


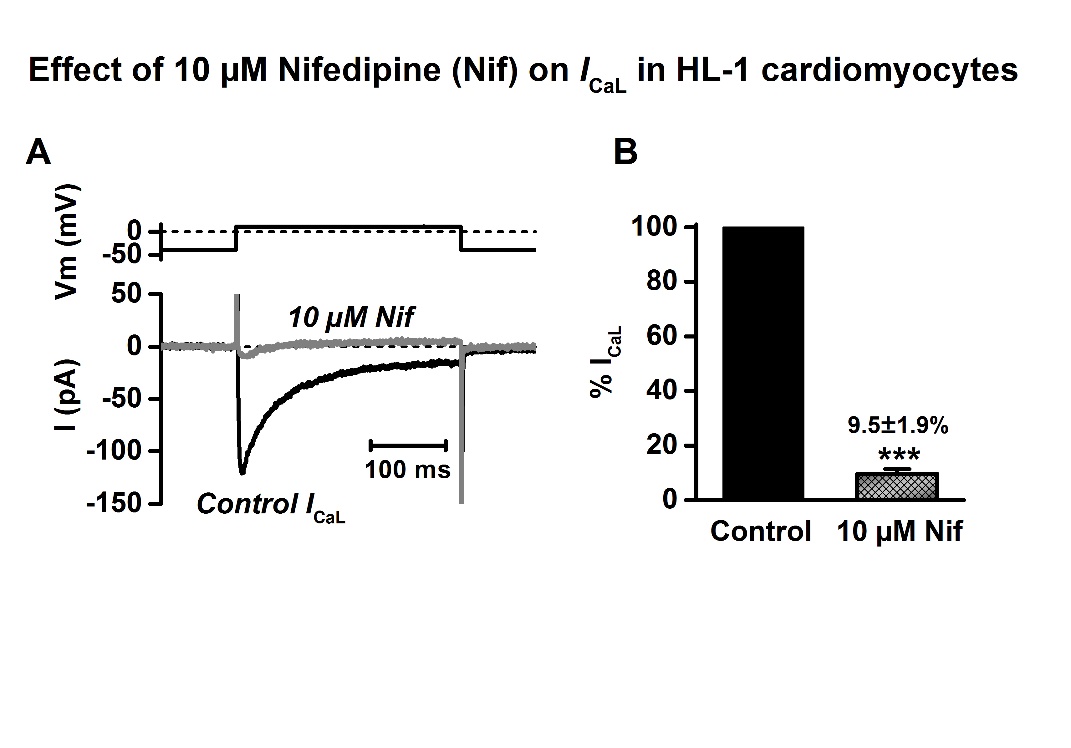


**Supplementary Figure 1:** Effect of Nifedipine on the L-type Ca^2+^ channels in HL-1 cardiomyocytes. **A)** Stimulation protocol and sample traces of control I_Ca, L_ (black trace) and during maximal inhibition with 10 µM nifedipine (Nif, grey trace) at +10 mV test potential.  **B)** Percentage of I_Ca, L_ inhibition by 10 µM Nif. Current is significantly reduced to 9.5+1.9 % of maximal I_Ca, L_ (*n*=5 cells of 3 cultures).
